# Comparison of Deep-Learning-Based Reconstruction of Knee MRI with Conventional Sequences in Patients with Knee Pain

**DOI:** 10.3390/diagnostics16182957

**Published:** 2026-09-13

**Authors:** Rola Husain, Stephen Belmustakov, Etan Dayan, Guillermo Carbonell, Ajit Shankaranarayanan, Mingqian Huang, Idoia Corcuera-Solano

**Affiliations:** 1 Diagnostic, Molecular and Interventional Radiology, Mount Sinai Health System, New York, NY 10029, USA; stephenbel70@gmail.com (S.B.); exd864@miami.edu (E.D.); guilleclc@gmail.com (G.C.); mingqian.huang@mountsinai.org (M.H.); idoia.corcuera-solano@mountsinai.org (I.C.-S.); 2 Dubai Hospital, Dubai Health, Muhammed Bin Rashid University of Medicine and Health Sciences, Dubai P.O. Box 505055, United Arab Emirates; 3 Subtle Medical Inc., 883 Santa Cruz Ave, Menlo Park, CA 94025, USA; ajit@subtlemedical.com

**Keywords:** deep learning-based reconstruction (DLR), subtle, knee, acquisition time

## Abstract

**Background:** Knee MRI is one of the most performed MR exams in outpatient settings and has a crucial role in detecting pathology. To increase access to advanced imaging and provide valuable diagnostic information to the clinician, reducing imaging acquisition time while preserving good image qualities is crucial. Deep learning-based reconstruction (DLR) algorithm (SubtleMR™) may be an alternative to conventional MRI with reduced scanning time while preserving image quality and diagnostic value. To compare image quality and diagnostic performance among conventional sequences and an automated accelerated deep learning-based reconstruction (DLR) knee algorithm method in patients with knee pain. **Material and Methods:** A total of 50 consecutive patients (26 men, mean age of 43.5 ± 10 years) with knee pain who underwent conventional (acquisition time: 13 min and 27 s) and accelerated DLR (acquisition time: 6 min and 45 s) knee MRI between November 2020 and December 2020 on a 1.5 T MRI system were prospectively enrolled. Two independent musculoskeletal radiologists compared the image quality artifacts and pathology using a 3-point Likert scale (1–3). Data were compared using paired Wilcoxon signed rank tests. **Results:** Forty-three patients with 86 knee MRI examinations were included. A comparison of conventional sequences and accelerated DLR by both readers revealed that there were no significant differences in overall diagnosis, image quality, and artifacts. Accelerated DLR knee MRI is comparable with conventional acquisitions, with an average time difference of 6 min and 42 s per patient (49.81%). **Conclusions:** Accelerated DLR knee MRI substantially reduces acquisition time (~49.81%) while maintaining equivalent diagnosis and image quality.

## 1. Introduction

Magnetic resonance imaging (MRI) of the knee is one of the most frequently performed examinations in musculoskeletal imaging. It is the preferred modality to assess osseous and soft tissue structures due to its superior soft tissue contrast resolution and multiplanar capability [1]. Knee MRI is commonly used to evaluate both traumatic and atraumatic knee pain, including meniscal pathology, cruciate ligament tears, and cartilage abnormalities, and to evaluate oncology cases of bone and soft tissue tumors [2]. However, the scan time of the conventional knee MRI technique limits the number of examinations performed per day, presents a challenge to some patients who are unable to stay still secondary to pain and discomfort, and exacerbates patient discomfort and anxiety that could potentially lead to motion-related artifacts or incomplete examinations. For these reasons, further research is necessary to achieve operational efficiency while maintaining excellent image quality with comparable diagnostic value. Artificial intelligence (AI) algorithms, and in particular, deep learning reconstruction (DLR) algorithms provide new ways for the detection, segmentation, and classification of imaging data sets and have been applied to knee MRI, primarily for the purpose of enhancing or automating diagnostic performance [3,4,5,6,7]. In addition, DLR has shown great potential for scan time reduction. However, the use of the DLR method for accelerated clinical knee MRI has yet to be extensively evaluated in the clinical setting. SubtleMR™ is an FDA-approved convolutional neural network-based DLR algorithm that allows for a faster acquisition using two methods of image enhancement: super-resolution and denoising. Images are sent from the MR scanner and are processed by SubtleMR™ software within seconds [8]. The high-quality images are then sent back to PACS and/or other workstations [9]. This application can be applied to all types of MR scanners.

The purpose of this study is to evaluate the image quality, diagnosis value, and potential artifacts of the new proposed DLR-MRI protocol in comparison with equivalent c-MRI protocol in the imaging of the knee joint while comparing their acquisition times.

## 2. Materials and Methods

### 2.1. Participants

This prospective single-center study followed the Health Insurance Portability and Accountability Act guidelines, and its protocol was approved by the institutional review board. No consent was obtained from subjects as per our local institutional review board protocol. However, all data were handled in compliance with the HIPPA guidelines. The inclusion criterion for this study was patients with a positive history for a clinical indication of knee abnormality that requires a routine knee MRI protocol.

A total of 50 consecutive outpatients undergoing routine knee MR imaging between November 2020 and December 2020 were included in our study and scanned with both conventional MRI and DLR-MRI protocols consecutively. Twenty-six men and twenty-four women with a mean age of 43.4 years (range, 13–78 years) were prospectively enrolled in this intra-individual comparative study. Patients underwent knee MR imaging examinations for a variety of clinical purposes; the clinical indication were pain (n = 30), pain and another indication [+effusion (n = 3), swelling (n = 2), trauma (n = 2), suspected osteochondritis dissecans (n = 1)], and internal derangement [general internal derangement (n = 2), anterior cruciate ligament tear (n = 5), collateral ligament sprain (n = 1), patellofemoral ligament sprain (n = 1), meniscal tear (n = 3), and bone-related pathology (n = 1)].

Out of the original 50 patients, 4 patients were excluded from the study due to a technical error, as their data were lost during the DL processing. An additional 3 patients with indwelling hardware were excluded from the study since the protocol for these patients differed from the routine sequences. Three axial reformats were excluded from the qualitative analysis due to a technologist-related error, where axial PD sequences were obtained in these studies instead of axial PD FS.

In total, we analyzed 86 examination sets in 43 patients, which included sagittal T2, coronal T2, and coronal PD FS, and 80 sets of axial PD FS.

### 2.2. DL Processing

DL processing of the scan data set was performed offline using an FDA-cleared convolutional neural network-based, deep learning image enhancement product, SubtleMR™ (Version 1.3, Subtle Medical, Menlo Park, CA, USA), with reported benefits including noise reduction and structure preservation. The knee DLR model does not directly remove imaging artifacts or enhance image sharpness. The DLR model is trained on thousands of multivendor MR datasets, at a variety of field strengths, vendors, clinical sites, and clinical indications, thus experiencing a range of tissue contrasts, acquisition parameters, patient anatomies, and variable image quality. Processing does not require proprietary raw k-space input (DICOM based) and is thus vendor-agnostic. DLR processing time per series is typically under a minute after the image acquisition on a graphical processing unit server connected to the scanner [9,10,11].

### 2.3. MRI Protocol

All MRIs were performed using a 1.5 T MR imaging system (Siemens Magnetom Altea and Amira, Erlangen, Germany) with an 18-channel knee coil. Section locations were identical across comparison scans. MR imaging data acquisition parameters are summarized in Table 1. Each patient was scanned two times during the same examination. Two sets of images were acquired consecutively for each scheme, one with conventional and another one with DLR algorithm (SubtleMR™ Version 1.3, Subtle Medical, Menlo Park, CA, USA) following our routine standard of care protocol. Each set of MR imaging included 5 sequences protocol which is the standard and most widely used MRI protocol for the study of the knee.

Axial PD FS, sagittal T2 FS, coronal T2 FS, and coronal PD images were obtained in both conventional-MRI and DLR-MRI protocols while sagittal PD images were only obtained with the conventional-MRI. The sagittal PD sequence, which is part of our conventional knee MRI protocol, was not included in the analysis since the speed gain is not possible with NEX = 1. Sagittal PD is acquired at an average NEX of 1, which is the lowest NEX number you can have as you must acquire the data at least once.

The scan time for the conventional sequence was 13 min and 27 s, and that for the accelerated sequence was 6 min and 45 s (a 49.81% reduction in time).

### 2.4. Qualitative Image Analysis

Two musculoskeletal radiologists (E.D. and M.H. with more than 5 and more than 15 years of subspecialty experience) independently performed qualitative image quality analysis and knee MRI anatomy evaluation. The readers were blinded to the sequence reconstruction method, radiologic reports, and pathologic information.

A total of 344 image sets from 86 examinations were evaluated for qualitative image quality analysis by both radiologists. The readers performed a blinded, de-identified side-by-side comparison of image sets to assess the overall image quality of predefined anatomical references to analyze the same anatomical structures.

**Figure 1:**
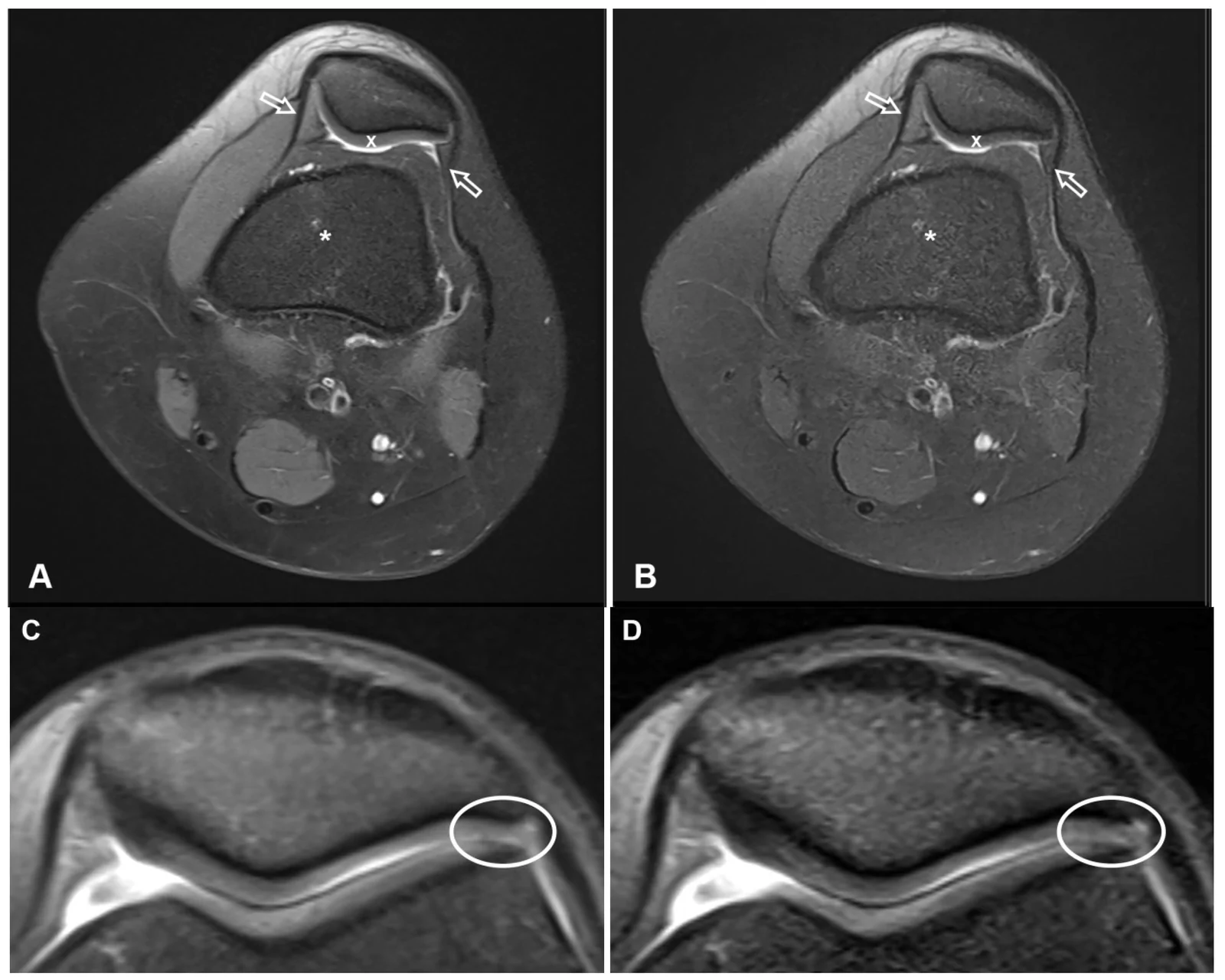
Axial PD FS. Conventional (**A**) vs. DLR (**B**) images, delineating the normal anatomy of the patellofemoral ligament (arrows), bone marrow (asterisk), and the cartilage (X). Conventional (**C**) vs. DLR (**D**) depicts minimal surface fibrillation of the lateral articular cartilage (circle).

**Figure 2:**
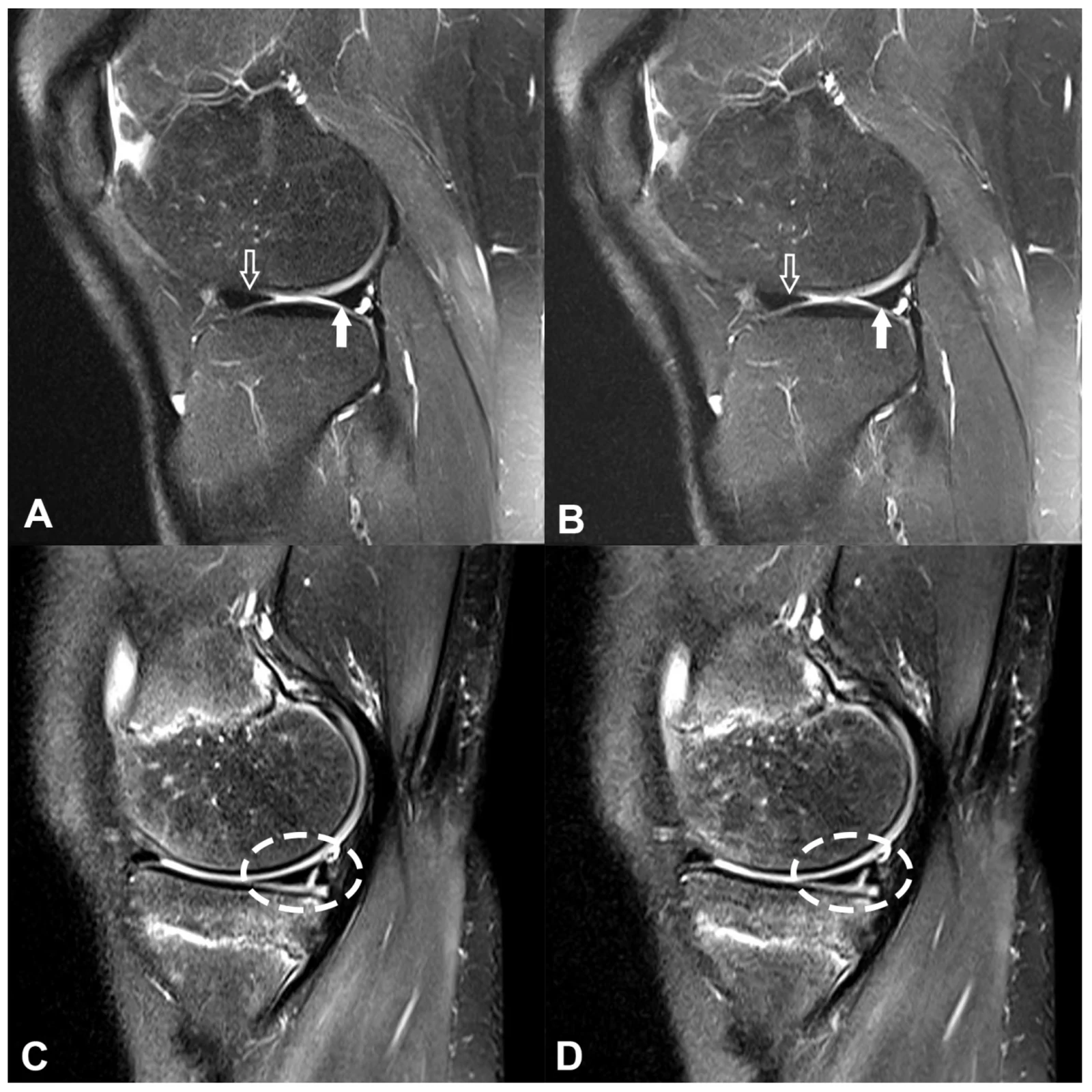
Sagittal T2 FS. Conventional (**A**,**C**) vs. DLR (**B**,**D**) images, illustrating the normal lateral meniscus: (**A**,**B**): anterior horn (void arrow) and posterior horn (white arrow), while (**C**,**D**) delineates a longitudinal tear involving the posterior horn of the medial meniscus (dotted oval).

**Figure 3:**
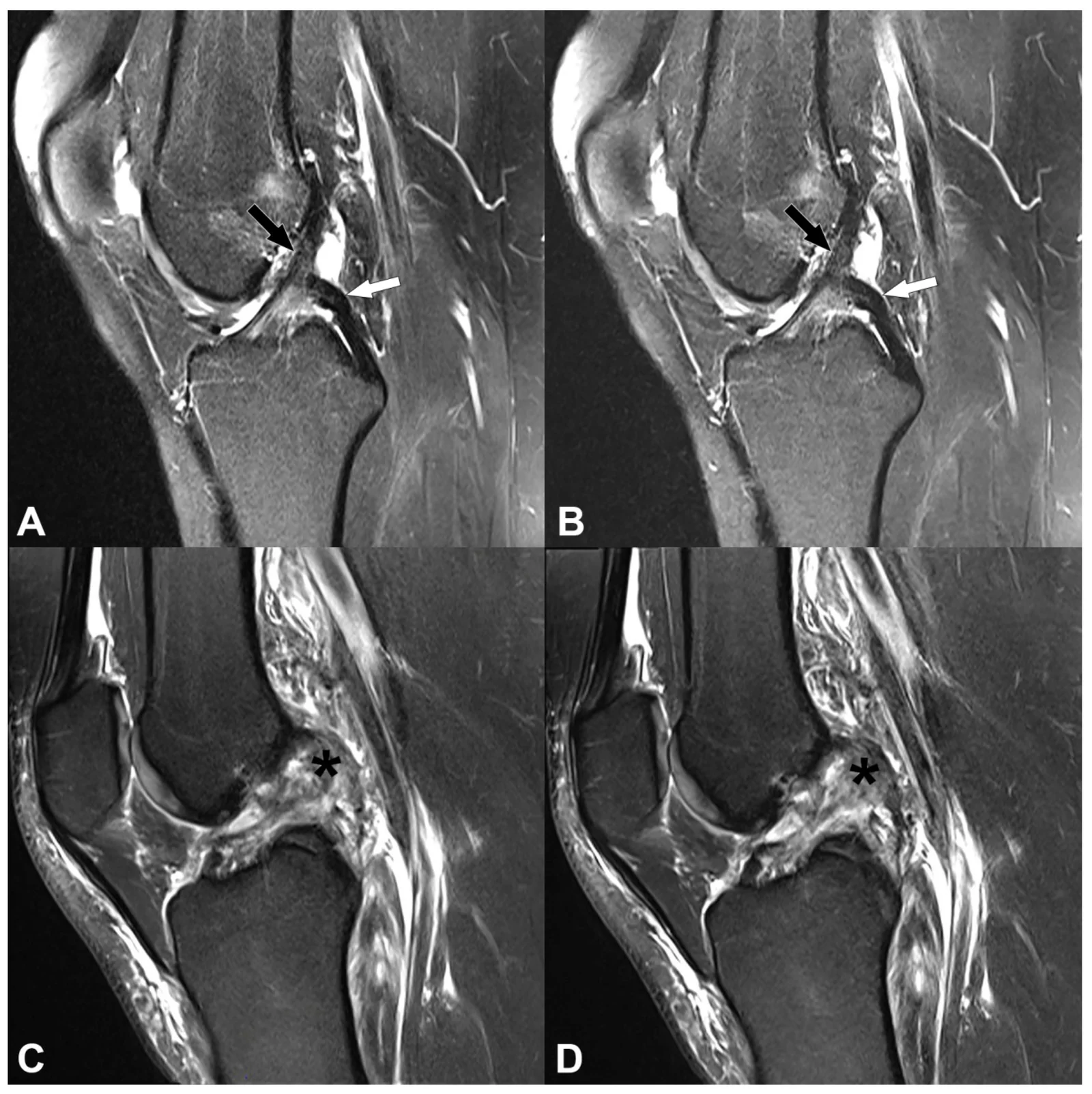
Sagittal T2 FS. Conventional (**A**,**C**) vs. DLR (**B**,**D**) images showing the normal anatomy (**A**,**B**) of the anterior cruciate ligament (black arrow) and the posterior cruciate ligament (white arrow), while (**C**,**D**) shows a completely torn anterior cruciate ligament (black asterisk).

**Figure 4:**
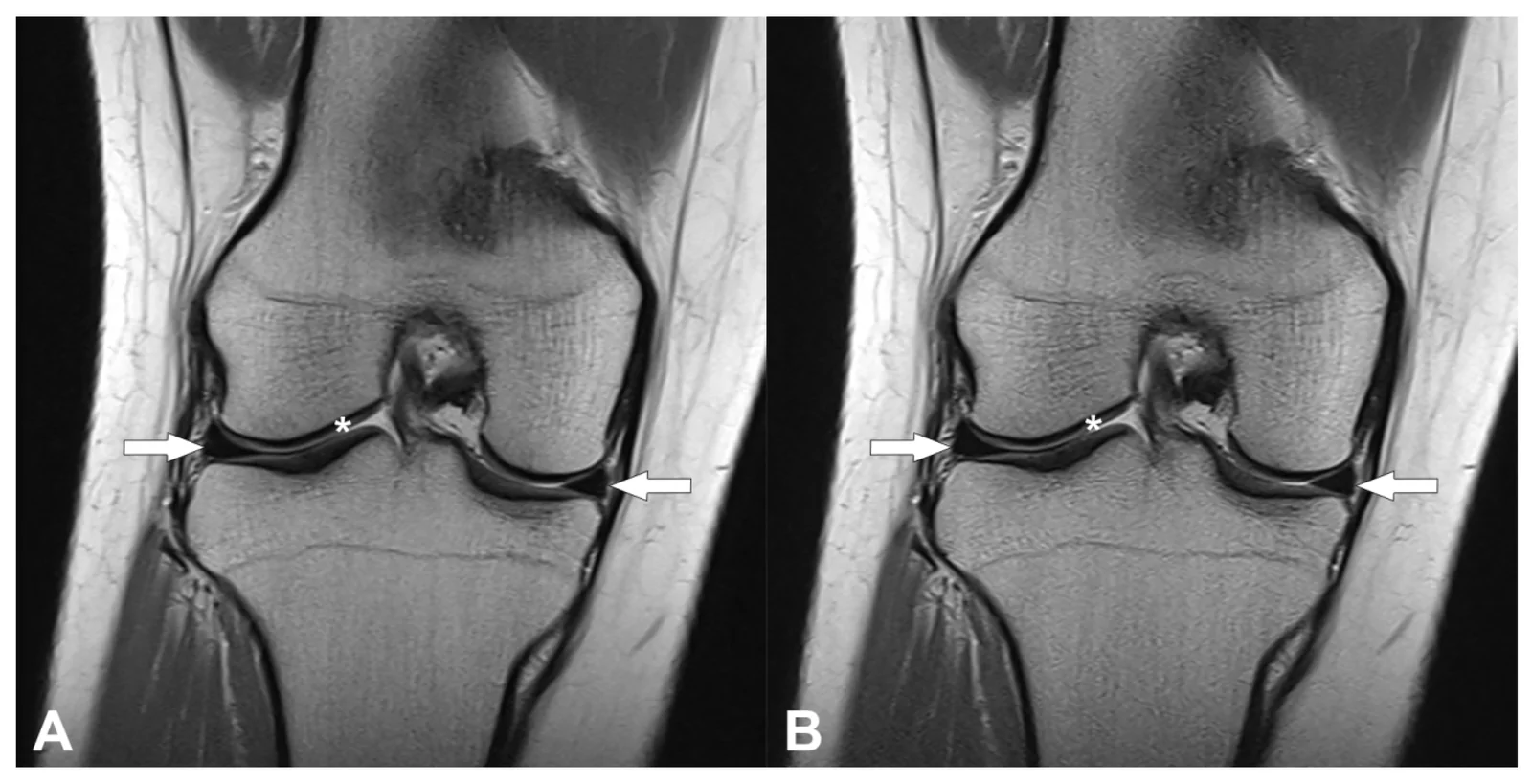
Coronal PD. Conventional (**A**) vs. DLR (**B**) images, illustrating the menisci (white arrows) and the cartilage (asterisk).

**Figure 5:**
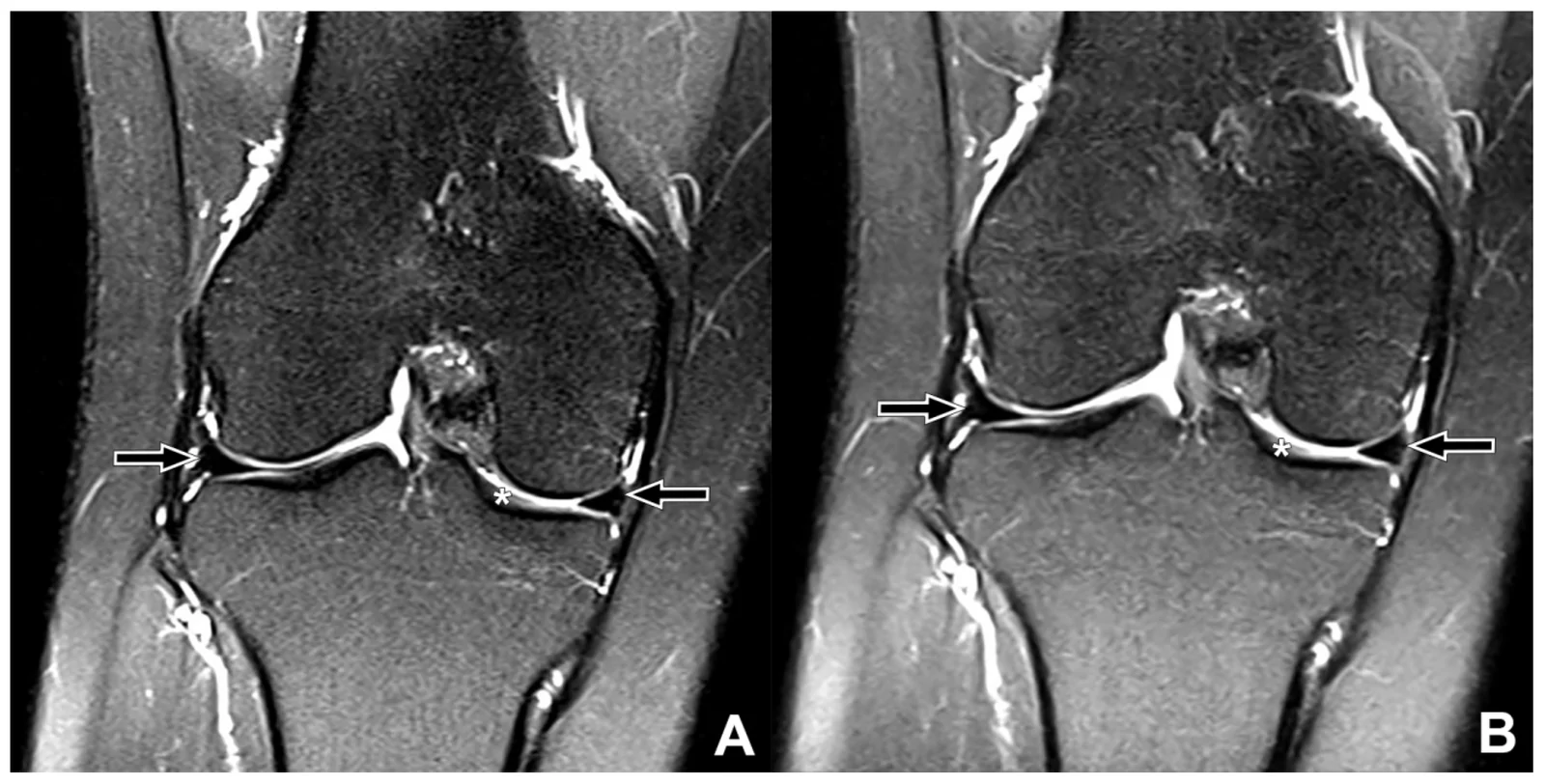
Coronal T2 FS. Conventional (**A**) vs. DLR (**B**) images, illustrating the menisci (black arrows) and the cartilage (asterisk).

A qualitative comparison of images acquired with DLR, and conventional approaches was performed. A total of 86 matched image sets were gathered from 43 patients with the following sequences axial PD FS (Figure 1A–D), sagittal T2 FS (Figure 2A–D and Figure 3A–D), coronal PD (Figure 4A–B), and coronal T2 FS (Figure 5A–B). The anatomical structures evaluated were the cartilage, patellofemoral ligament, bone marrow, menisci, and cruciate ligaments, as well as the diagnostic value.

All image sets were presented interchanging the order of conventional as well as DLR sequences in a random de-identified manner and were evaluated on a viewing workstation (Advantage Workstation 4.6; GE Healthcare, Chicago, IL, USA), at the same time. The rationale behind the side-by-side comparison was to evaluate the images at the same slice on both modalities (DLR and conventional). For the image quality analysis, each image was independently evaluated, utilizing a 3-point Likert scale: 1 being worse than expected (in comparison to a routine-encountered sequence), 2 being as expected, and 3 being better than expected. Readers assessed the overall sharpness and subjective SNR of the following anatomical structures: cartilage, patellofemoral ligament, bone marrow, menisci, and cruciate ligaments [12]. In addition, readers evaluated each image independently for the presence of artifacts using a 3-point Likert scale: 1 severe artifact, 2 minimal artifacts, and 3 no artifact. Finally, the diagnosis was also evaluated in each image independently, using a 3-point Likert scale to assess for internal derangement (meniscal tears, ligament, tendons abnormalities, chondral defects, and bone marrow signal-intensity abnormalities) comparing both image sets. For the Likert scale, 1 was non-diagnostic, 2 was equivocal, and 3 diagnostic. Afterward, the 3-point Likert scale results and readers’ preferences of each set were compared and recorded.

### 2.5. Statistical Analysis

A nonparametric paired Wilcoxon test was used to compare the qualitative parameters between the DLR and conventional image sets pairs, to test the null hypothesis that there was no significant difference between DLR-MRI and conventional-MRI. Image quality scores for each set of MR images were analyzed. Qualitative results are expressed as mean and standard deviation. Statistical analysis was performed by using commercially available software (SPSS, Version 20; IBM, Armonk, New York, NY, USA). A two-tailed *p* value of less than 0.05 was considered to indicate statistical significance (Table 2).

## 3. Results

### 3.1. Acquisition Time

The average acquisition time reduction for the study of the knee MRI between conventional and DLR protocols (axial PD FS, sagittal T2 FS, coronal T2 FS, and coronal PD) was found to be 6 min and 42 s per patient (49.81%reduction). The average acquisition time with the use of the DLR algorithm was 6 min 45 s vs. 13 min and 27 s with the conventional MRI acquisition.

The difference between conventional sequences with their equivalent DLR sequences was 2 min and 6 s (60.0% reduction) for the axial PD sequence, 1 min and 54 s (47.3% reduction) for the sagittal T2 FS sequence, 1 min and 9 s (46.3% reduction) for coronal sequence, and 1 min and 33 s (44.9% reduction) for coronal T2 FS sequence. The average acquisition time for sagittal PD was 2 min and 58 s (Table 3).

### 3.2. Qualitative Analysis

#### 3.2.1. Diagnostic Value Analysis

No significant differences were noted between DLR-MRI and conventional-MRI when assessing internal derangement on axial PD FS, sagittal T2FS, coronal PD, or coronal T2FS sequences by any reader. Although several individual image-quality parameters reached statistical significance, all average image-quality scores remained above 2 on the 3-point Likert scale, corresponding to images considered diagnostically acceptable by experienced musculoskeletal radiologists. Importantly, no statistically significant differences were identified in overall diagnostic performance or diagnostic confidence between conventional-MRI and DLR-MRI.

Therefore, while subtle differences in image quality were measurable for selected anatomical structures, these differences did not translate into clinically meaningful differences in diagnosis.

#### 3.2.2. Anatomical Analysis

When analyzing the performance of each sequence assessing overall image quality (all anatomic structures added for the statistical analysis), significant differences were found on axial PD FS for both readers (*p* < 0.002) and coronal T2 for reader 1 (*p* = 0.004), where c-MRI demonstrated superior results that DLR. Specifically, regarding preselected anatomic structures, no significant differences in image quality were noted on axial PD FS, sagittal T2, coronal T2, or coronal PD for any reader when assessing patellofemoral ligament, menisci, and cruciate ligaments (*p* > 0.05). DLR images demonstrated a significantly higher score for the assessment of the cartilage on coronal PD when compared to the equivalent conventional sequence for reader 1 (*p* < 0.008). However, DLR images demonstrated a significantly lower score when compared to equivalent conventional sequence on reader 1 when assessing cartilage on axial PD FS (*p* < 0.001) and when assessing bone marrow on axial PD FS (*p* < 0.001), sagittal T2 (*p* < 0.001), and coronal T2 (*p* < 0.001). Nevertheless, all DLR images were rated with a score above 2 in all sequences meaning the image quality of these structures were “as expected” by a musculoskeletal radiologist.

#### 3.2.3. Artifact Analysis

When the artifact was assessed, no significant differences in artifact were noted in sagittal T2, coronal T2, or coronal PD for any reader. However, DLR axial PD FS images demonstrated a perceived increase in artifacts with a significantly lower score when compared to equivalent conventional sequences for both readers (*p* < 0.008). Table 4 summarizes the statistical results.

## 4. Discussion

**Figure 6:**
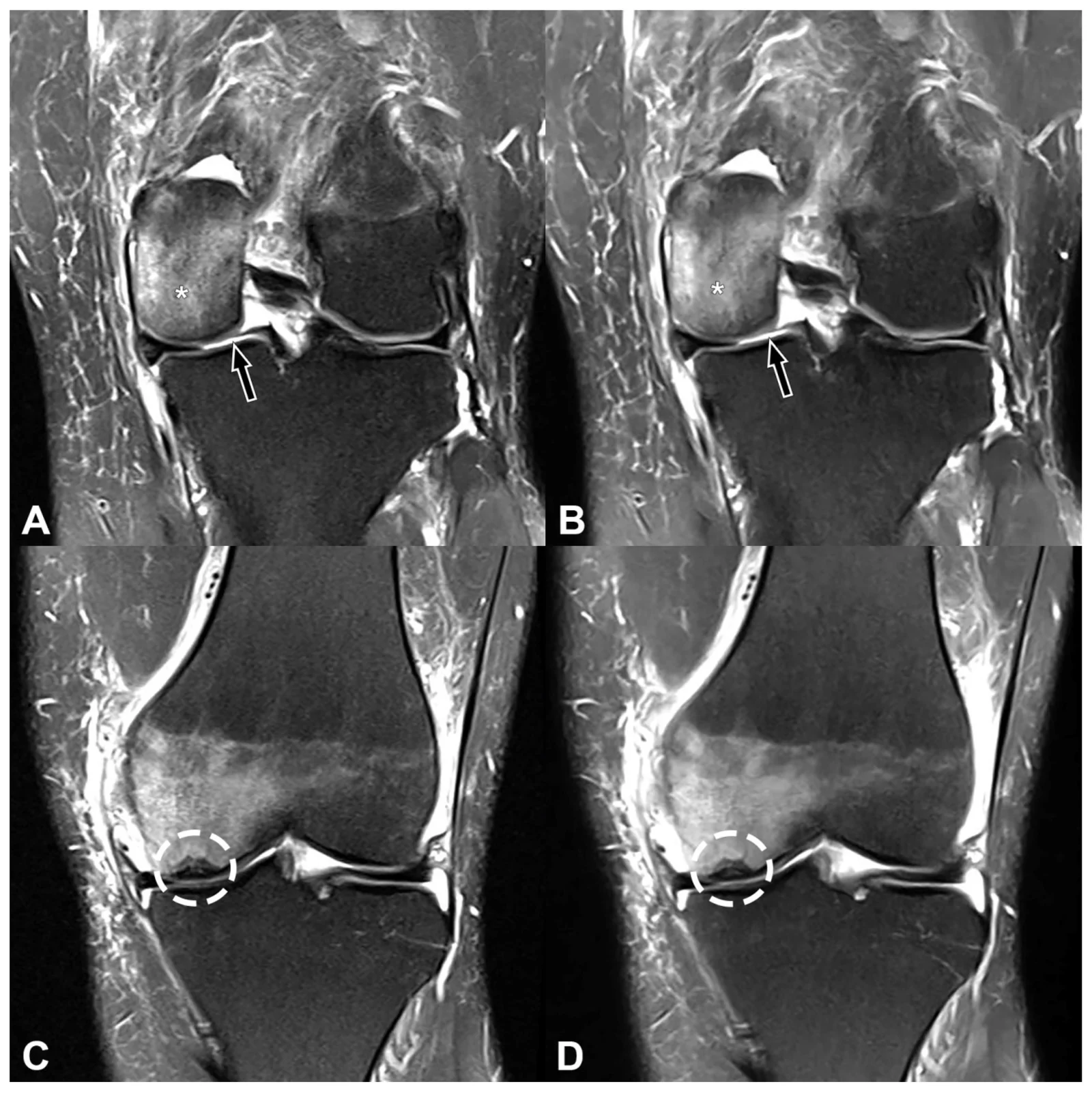
Coronal T2 FS. Conventional (**A**,**C**) vs. DLR (**B**,**D**) images, illustrating a high-grade radial tear-free margin of the posterior medial horn (black arrow), along with a non-depressed subchondral insufficiency fracture (dotted circle) with associated moderate reactive marrow edema involving the medial condyle (white asterisk).

MRI is the best image modality to assess knee pathology and is one of the most performed MR exams, especially in the outpatient setting. However, routine scanning protocols for knee MRI can take considerable time. Rapid MR evaluations are important to increase access to advanced imaging for all patients and at the same time provide rapid valuable diagnostic information to the clinician. Therefore, decreasing imaging acquisition time has been a major ongoing field of research since the invention of MRI. Earlier techniques to shorten MR scanning times resorted to parallel imaging, which allows simultaneous acquisition of some data points using multi-element detector arrays [13,14,15,16], and compressed sensing, which gathers a reduced set of data points and searches for the most compressed image that is consistent with those data [16,17,18]. Both methods achieve accelerated acquisitions by sub-sampling k-space and, while these speeds up the acquisition because less data need to be collected, it also introduces artifacts in the reconstructed image. A new DLR has been proposed as a means of solving the problem of reconstructing MR images from the accelerated acquisitions [19]. DLR methods for reconstructing MR images from under-sampled data can learn from images of significantly higher complexity than those used for compressed sensing and therefore may allow previously inaccessible levels of acceleration while preserving high image quality. Early studies have shown promising results in terms of image quality and diagnostic accuracy when DL accelerated knee MRI is compared to conventional MR [20]. In our study, we compared conventional MRI protocol to an alternative recently proposed DLR image for accelerated MR examination of the knee using SubtleMR™ software in patients being evaluated at 1.5 T MRI. Image quality, artifacts, and overall diagnosis were compared across multiple sequences and knee pathology. To our knowledge, this is the first report in the literature assessing the performance of SubtleMR™ DLR-assisted imaging acquisition using a complete knee MR imaging protocol in the clinical setting, rather than isolated sequences or phantom data [21,22,23,24]. Our study included common etiology of knee pathology such as meniscal and ligamentous injuries, cartilage loss, and contusions (Figure 2, Figure 3 and Figure 6).

The results of our study show overall very good concordance between the current clinical gold standard, conventional-MRI, and our accelerated DLR-MRI protocol, with substantially reduced acquisition time. DLR-MRI images demonstrated equivalent overall diagnostic performance to conventional-MRI images and, though ratings in regards to image quality varied among both readers, the average score in all cases was above 2, implying that DLR imaging quality in all cases was of diagnostic value by a musculoskeletal radiologist. In most cases, no statistically significant differences were found amongst DLR-MRI and conventional-MRI sequences; however, few criteria were rated higher on conventional imaging. For reader 1, there was a perceived lower image quality of bone marrow on T2-weighted sequences and of cartilage on coronal PD, and for both readers, there was a perceived increase in artifact on axial PD FS images. One potential reason for perceived increased artifacts on DLR-MRI axial PD FS images could results from the fact that we tried to accelerate that sequence more than the others (NEX 3 → 1 vs. NEX 2 → 1), additionally DLR-MRI studies were always acquired after the conventional imaging and that the axial PD FS sequence was the last one to be acquired. This may also have contributed to motion artifacts toward the end of the examination.

The image acquisition time of a standard non-contrast knee MRI is about 18 min [25]. In our institution, 40 min are allocated as a standard of care for a routine knee MRI study using our conventional acquisition protocol and accounting for patient screening, set up, and cleaning before and afterward; of those, approximately 20 min is the imaging acquisition time (not taking into consideration for any claustrophobic or uncooperative patients, which may lead to repeat acquisitions to address motion artifacts). The average acquisition time reduction between conventional and DLR protocols was found to be 6 min and 42 s per patient, which represents a 49.81% reduction. The use of DLR-MRI acquisition protocol could ease the workflow leading to increased productivity. In our institution, our outpatient MRI suite operates 13 h a day. With 40-min blocks allocated per patient for a standard knee MRI exam, up to 19 patients can be scheduled a day in a single MRI scanner. With the use of DLR-MRI, we would be able to accommodate up to 39 patients given the reduced scan time, thus significantly increasing patient access. This technology could be applied to any MR scanner at a variety of field strengths and theoretically to any anatomic region. Prior research data from Subtle in the spine and brain studies demonstrated similar results, achieving up to 30–50% exam time reduction while maintaining image quality [13,25,26].

Although various prior studies have investigated the use of DLR in MR images [27,28,29,30,31], very few studies have assessed knee pathology [32,33].

We acknowledge several limitations in our study. First, DLR-MRI was always acquired after the c-MRI protocol was completed, potentially penalizing DLR imaging quality due to more potential motion artifacts as patients had already been in the MRI suite for 20 min. Sconed, the study population was relatively small. however, this study was intended as a proof-of-concept clinical validation; hence the investigation was designed as a prospective intra-individual comparative study, in which each patient underwent both conventional MRI and DLP MRI, in the same setting, serving as their own control, rendering the study rather unique, contributing to reduced inter-patient variability. Nevertheless, the absence of an independent reference standard serves as an additional third limitation. Fourth, only patients undergoing routine knee MRI protocols were included in the study as we needed sufficient patient data with the matching sequences to compare, lacking post-contrast MRI studies such as oncology cases and postsurgical cases requiring metal reduction techniques. Fifth, the absence of a comparison to compressed sensing or the use of a real reconstruction methodology impacts the study; considering that SubtleMR is not a reconstruction method as it uses image output from MRI scanner, hence further research is suggested in this area, comparing SubtleMR to other image denoising methods. Sixth, we note the absence of comparison between the quality of NEX = 1 images and SubtleMR results, lacking crucial evidence of added benefit of SubtleMR in increasing image quality. Moreover, we acknowledge that a thorough scoring system (such as 5-point Likert scale) may serve better to detect subtle differences between reconstruction methods; hence, this may create a limitation to our study. Lastly, this preliminary study compared c-MRI and DLR-MRI on a single MRI system. However, we have provided our imaging parameter choices that can translate to other scanners. In a future study, it would be helpful to compare the sequences on different systems from various vendors and different field of strength as DLR-MRI is vendor-independent, considering that our study was conducted utilizing 1.5 Tesla in a single center.

## 5. Conclusions

In conclusion, our study demonstrated that DLR-MRI can produce images of diagnostic value compared to conventional-MRI while significantly decreasing the acquisition time in knee MRI imaging. This could improve patients comfort through shorter examination time, reduced probability of motion artifacts during image acquisition, and consequently increase MR scanner output, improving the workflow by improving scheduling capacity with a potential reduction in healthcare costs associated with increased examination time. This will be rather useful in pediatric settings, claustrophobic individuals, elderly patients, or patients with severe pain that prevent them from remaining still for prolonged MR examinations. Our findings suggest that DLR-MRI techniques may represent a potential viable alternative to conventional-MRI in clinical practice for the evaluation of knee pathology, allowing for a substantial reduction in acquisition time (49.81%); however, considering our limitations, and in particular the single center study with small study size, further research in this area is needed to explore and consolidate these results.

## Figures and Tables

**Table 1:** Summary of MR imaging parameters.

	Conventional-AX PD-Weighted FS	DLR-AX PD-Weighted FS	Conventional-SAG T2-Weighted FS	DLR-SAG T2-Weighted FS	Conventional-COR PD-Weighted	DLR-COR PD-Weighted	Conventional-COR T2-Weighted FS	DLR-COR T2-Weighted FS
**TR (ms)**	3700	3540	6490	6490	3500	3500	6280	6370
**TE (ms)**	27	27	71	71	36	36	64	64
**FOV (cm)**	15	15	15	15	15	15	15	15
**Matrix**	258 × 416	258 × 416	224 × 320	224 × 320	258 × 416	258 × 416	224 × 320	224 × 320
**Section thickness (mm)**	3.5	3.5	3.5	3.5	3.5	3.5	3.5	3.5
**Section spacing (mm)**	0	0	0	0	0	0	0	0
**No. of sections**	44	44	40	40	40	40	40	40
**Bandwidth (kHz)**	131	131	130	130	160	160	140	140
**Refocus flip angle**	180	180	150	150	180	180	150	150
**Echo-train length**	17	17	18	19	17	17	15	16
**NEX**	3	1	2	1	2	1	2	1
**Acceleration factor**	2	2	2	2	2	2	2	2
**Scan time (min)**	3:23	1:14	4:02	2:11	2:12	1:12	3:16	1:50

**Table 2:** Image quality scores for conventional sequences in different planes and equivalent DLR sequences from two independent readers.

		Reader 1			Reader 2		
		Conventional	DLR	*p*	Conventional	DLR	*p*
**Axial (40)**	**Cartilage**	2.96 ± 0.223	2.54 ± 0.56	0.00	3.00 ± 0.00	2.90 ± 0.31	0.05
	**PFL**	3.00 ± 0.00	2.97 ± 0.16	0.32	3.00 ± 0.00	3.00 ± 0.00	1.00
	**Bone Marrow**	2.92 ± 0.27	2.31 ± 0.57	0.00	3.00 ± 0.00	3.00 ± 0.00	1.00
	**Artifact**	2.56 ± 0.50	2.38 ± 0.54	0.00	2.95 ± 0.22	2.77 ± 0.43	0.01
	**Diagnostic**	3.00 ± 0.00	2.96 ± 0.27	0.08	3.00 ± 0.00	3.00 ± 0.00	1.00
**Sagittal T2 (43)**	**Menisci**	2.98 ± 0.15	3.00 ± 0.00	0.32	3.00 ± 0.00	3.00 ± 0.00	1.00
	**Cruciate**	2.98 ±.15	2.98 ±.15	1.00	3.00 ± 0.00	3.00 ± 0.00	1.00
	**Bone Marrow**	2.93 ± 0.26	2.57 ± 0.50	0.00	2.95 ± 0.22	3.00 ± 0.00	0.16
	**Artifact**	2.31 ± 0.52	2.4 ± 0.50	0.21	2.67± 0.57	2.74 ± 0.45	0.32
	**Diagnostic**	2.95 ± 0.22	3.00 ± 0.00	0.16	2.98 ± 0.15	3.00 ± 0.00	0.32
**Coronal PD (43)**	**Menisci**	2.93 ±.26	3.00 ± 0.00	0.08	2.98 ± 0.15	3.00 ± 0.00	0.32
	**Cartilage**	2.88 ± 0.38	3.00 ± 0.00	0.01	2.98 ± 0.15	3.00 ± 0.00	0.32
	**Bone Marrow**	2.90 ± 0.0	2.98 ± 0.15	0.18	2.98 ± 0.15	3.00 ± 0.00	0.32
	**Artifact**	2.95 ± 0.22	3.00 ± 0.00	0.16	2.93 ± 0.34	2.98 ± 0.15	0.41
**Coronal T2 (43)**	**Menisci**	2.95 ± 0.22	2.90 ± 0.30	0.41	2.95 ± 0.31	2.98 ± 0.15	0.66
	**Cartilage**	2.88 ± 0.33	2.79 ± 0.47	0.25	2.93 ± 0.34	2.93 ± 0.34	1.00
	**Bone Marrow**	2.88 ± 0.33	2.43 ± 0.55	0.00	2.93 ± 0.26	2.93 ± 0.26	1.00
	**Artifact**	2.81 ± 0.51	2.71 ± 0.51	0.43	2.83 ± 0.44	2.88 ± 0.40	0.59
	**Diagnostic**	2.85 ± 0.22	2.95 ± 0.31	1.00	3.00 ± 0.00	3.00 ± 0.00	1.00

**Table 3:** Comparison of time difference.

	Conventional	DLR	Difference
**AX PD FS**	3:30	1:24	2:06
**Sag T2FS**	4:01	2:07	1:54
**Cor PD**	2:29	1:20	1:09
**Cor T2 FS**	3:27	1:54	1:33
**Total study time**	13:27	6:45	6:42

**Table 4:** Sequences preference between the two readers.

Preference	READER 1			READER 2		
	Conventional	DLR	No Preference	Conventional	DLR	No Preference
**AX PD**	11	1	34	28	7	11
**Sag T2FS**	3	4	39	18	8	20
**Cor PD**	2	6	38	2	20	24
**Cor T2 FS**	3	7	36	32	3	11

## Data Availability

The original contributions presented in this study are included in the article. Further inquiries can be directed to the corresponding author.

## Abbreviations

MRI	Magnetic Resonance Imaging
DLR	Deep learning-based reconstruction
FDA	Food and Drug Administration
AI	Artificial Intelligence
HIPAA	Health Insurance Portability and Accountability Act
